# Exploring the future of robotic approaches in neonatal cardiac surgery: opportunities, barriers, and innovation pathways

**DOI:** 10.1007/s11701-025-03004-x

**Published:** 2025-11-29

**Authors:** Siena Martin, Mirkomol Mirzarakhimov, Omar Shafi, Mauro Camacho

**Affiliations:** 1 City St Georges Medical School, Cranmer Terrace, SW17 0RE London, England; 2 Brunel Medical School, UB8 3PH Uxbridge, England

**Keywords:** Robotic surgery, Neonatal cardiothoracic surgery, Congenital heart disease, Minimally invasive surgical intervention, Surgical innovation

## Abstract

Robotic-assisted surgery (RAS) emerged as a transformative approach in various surgical disciplines, particularly in adult cardiothoracic procedures, where it has demonstrated significant benefits such as enhanced precision, improved visualisation, and reduced complication rates. Despite its success in adult surgeries, including mitral valve repairs and coronary artery revascularisations, the application of RAS in neonatal cardiothoracic surgery remains largely unexplored. This narrative review synthesises existing evidence, highlighting the potential of RAS to address the unique challenges posed by neonatal cardiothoracic procedures, which require intricate manoeuvrability and precision within confined anatomical spaces. The review also examines the successes of minimally invasive techniques, such as laparoscopic and thoracoscopic surgeries, in paediatric patients, particularly those with congenital heart disease (CHD), demonstrating their safety and efficacy even in high-risk populations. These findings suggest that RAS could offer similar, if not superior, benefits in neonatal cardiothoracic surgeries in future. Significant gaps in the literature, particularly regarding long-term outcomes and the technical challenges of RAS adaptation for neonatal use, underscore the need for further research. Likewise, technological iterations in the currently available systems (including instrument size and curvature, scope definition and haptic feedback) are required to make this concept a reality by meeting the standard of care. This review argues that while RAS holds considerable promise for improving surgical outcomes in neonates, dedicated research is essential to overcome and fully realise its potential. This review not only underscores the current successes of RAS in adult and paediatric surgeries but also sets a foundation for future exploration into its application in neonatal cardiothoracic procedures, advocating for continued innovation and research in this promising field.

**Supplementary Information:**

The online version contains supplementary material available at 10.1007/s11701-025-03004-x.

## Introduction

Advances in medical and surgical technologies are driving a collective shift towards precision and atraumatic intervention [1–4]. From robotic surgery and nanofibre-based drug delivery to advanced imaging therapeutics and nano-engineered systems, these novel technologies aim to provide maximal specificity and minimal invasion: principles nowhere more pertinent than in the field of neonatal surgery [5]. Robotic-assisted surgery (RAS) is an emerging approach for neonatal congenital heart disease. Since the first robotic-assisted open-heart surgery in 1998 using an early da Vinci system, RAS has transformed many surgical fields, especially adult cardiothoracic surgery [6]. Enhanced 3D vision, superior dexterity, motion scaling, and tremor filtration have made it effective for procedures like mitral valve repair and coronary revascularisation [7]. Over time, these advantages have led to successful applications in selected paediatric populations, including adolescents and older children. Despite the broad adoption of RAS in adult and older paediatric cardiac surgery, its use in neonates remains virtually absent [8]. This is particularly striking given that congenital heart anomalies (CHAs), including septal defects, outflow tract obstructions, and valve malformations, often require surgical correction within the first weeks of life [9, 10]. Many of these anomalies are driven by perinatal factors such as prematurity, intrauterine growth restriction, and haemodynamic instability, which further increase the demand for early surgical intervention in neonates [11, 12]. While neonatal cardiothoracic surgery demands extreme precision within confined anatomical spaces, RAS could theoretically meet these demands better than traditional open or minimally invasive techniques. Emerging evidence from adult and adolescent populations supports the procedural feasibility and favourable outcomes of robotic cardiac surgery [7, 13]. High-volume centres report low complication and conversion rates for various intracardiac procedures, including congenital anomalies. Yet, the lack of neonatal cases underscores a major research gap amid the rising global prevalence of congenital heart disease (CHD) and the need for early-life surgical innovation [14–16].

Today, the RAS approach continues to provide benefit to cardiothoracic procedures in adults and older paediatric cases. A RAS approach might be chosen over a conventional laparoscopic approach for cardiothoracic procedures, on account of certain key benefits; increased degrees of freedom, tremor filtration, 3D visualisation, and motion scaling [17]. Indeed, recent work emerging from various robotic cardiac surgery programmes worldwide has successfully applied the RAS approach to a broad range of cardiac procedures in adults and older paediatric cases, including mitral valve (MV) procedures, coronary artery revascularisation (CAR), ASD repair and beyond [18]. Moreover, this broadening range of cases to which the RAS approach has been applied includes CHA repair in adults and older children. Onan and Onan’s study of 242 patients, with a mean age of 30.9 years, reported successful RAS treatment of conditions such as ASD, partial anomalous pulmonary venous connections (PAPVC), and ventricular septal defects (VSD), with minimal postoperative complications and no mortality [14].

It must be emphasised that a RAS approach to neonatal CHD repair is not yet possible. Today’s ergonomic limitations render the currently available systems inapplicable to the particularities of neonatal anatomy: systems would need to first evolve (including in terms of instrument size and curvature, scope definition and haptic feedback) to meet the standard of care. However, whilst early, research is emerging to describe early instances of an applied RAS approach to the neonatal population (including neonatal robotic fundoplications, gastrostomies, pyloroplasties, congenital diaphragmatic hernia repairs, nephrectomies and beyond) which might support the case for the necessary technological developments [19]. Moreover, current evidence of safe thoracoscopic and laparoscopic use in neonates with CHD suggests a viable foundation for robotic adaptation within the thoracic cavity, making this approach a worthwhile avenue for exploration [20, 21]. This narrative review explores robotic cardiac surgery and its potential in neonatal CHD. By integrating evidence from adult and paediatric cases and assessing current platform limitations, it outlines key translational challenges, present day barriers and future steps toward making neonatal robotic cardiac surgery feasible and effective in the future.

## Methods

- **Literature Search and Scope**.
  i. A broad literature search was undertaken to identify studies published between January 1994 and July 2024 relevant to robotic, laparoscopic, and thoracoscopic surgery in adult, paediatric, and neonatal populations. Databases included PubMed, MEDLINE, EMBASE, the Cochrane Library, and Google Scholar. Both controlled vocabulary and free-text terms were used to maximise coverage.
- **Study Selection**:
  i. Studies were included if they described:
    1. Design: cohort studies, randomised controlled trials (RCTs), meta-analyses, systematic reviews, narrative reviews, case reports, or editorials (the latter used for contextual insights).
    2. Population: adult, paediatric, or neonatal patients undergoing robotic, thoracoscopic, or laparoscopic cardiothoracic surgery, or paediatric abdominal procedures relevant to congenital cardiac disease.
    3. Intervention: robotic, thoracoscopic, or laparoscopic surgical techniques.
    4. Outcomes: safety, feasibility, surgical success, complication rates, or recovery measures.
  ii. Exclusion criteria were:
    1. Non-English language publications,
    2. Studies without clear outcome measures.
- **Approach to Evidence Appraisal**.
  i. Emphasis was placed on identifying the most relevant and influential work in the field, with close attention to representativeness, and reported outcomes.
- **Data Synthesis**.
  i. Findings were synthesised narratively and thematically. Evidence was organised around three domains:
    1. Procedural feasibility and outcomes in adult and adolescent populations,
    2. Tolerability of minimally invasive approaches in neonates,
    3. Anatomical and technological challenges unique to neonatal surgery.
    4. The intent was not to statistically pool data, but rather to integrate findings across diverse study types in order to highlight translational gaps and future directions.
- **Ethical Considerations**:
  i. In conducting this narrative review, ethical considerations were rigorously adhered to throughout the research process. As the review involved data from previously published studies, no new data was collected directly from human participants, thereby minimising ethical risks. However, all selected studies were carefully evaluated to ensure that they had obtained appropriate ethical approval and informed consent where required. Additionally, this review adhered to the principles of transparency and integrity in research, ensuring that all data was accurately reported, and no conflicts of interest were present. Furthermore, the review process was conducted with respect for the original authors’ intellectual property, appropriately crediting all sources and avoiding any form of plagiarism. These ethical practices were integral to maintaining the credibility and reliability of the narrative review.

## Background

### Evolution and success of the RAS approach to cardiothoracic procedures in adolescent and adult populations

This study builds on a rich body of work from robotic cardiac surgery programmes which have demonstrated the safety and efficacy of a RAS approach to cardiothoracic procedures in paediatric (primarily, adolescent) and adult populations. In cardiac surgery, across these populations, a RAS approach has historically been most widely used for MV surgery and CAR [22]. A RAS approach is estimated to account for 35% of all minimally invasive MV repairs [22]. The rationale for this tendency is better visualisation and enhanced dexterity within confined areas necessary in intracardiac procedures [23]. A review of short-term clinical outcomes for 997 patients (mean age, 56 years ± 10 years) who underwent robotic mitral valve surgery demonstrated success (reporting outcomes based on predischarge echocardiography) with 99.5% (992 patients) successfully receiving the repair and only 0.5% (5 patients) requiring valve replacement. Intraoperative echocardiography showed that 99.7% of patients had no or only mild mitral regurgitation immediately after surgery, a positive outcome maintained in 97.9% of patients at hospital discharge. The study also reported a very low hospital mortality rate of 0.1% (1 patient), although 1.4% (14 patients) experienced a stroke. Notably, the stroke rate improved from 2% in the initial 500 patients to 0.8% in the subsequent 500, reflecting procedural enhancements over time. Significant reductions were also observed in myocardial ischemic times, cardiopulmonary bypass times, blood transfusions, and postoperative intensive care unit and hospital stays [24]. Based on these findings, it was concluded that a RAS approach to MV surgery was associated with a high likelihood of success, low morbidity and low mortality. This success was attributed to improved visualisation and dexterity, afforded by a RAS approach. However, it is important to acknowledge that this study reported short-term outcomes, based on pre-discharge echocardiography findings, only [24].

In 2022, Balhky et al., at the University of Chicago Medicine Robotic and Minimally Invasive Cardiac Surgery Programme published a comprehensive review of all outcomes from more than 1,100 robotic cardiac procedures carried out between 2013 and 2021 to assess for mortality and perioperative morbidity [18]. Building on the work of Gillinov et al., [24], this study did not only look at MV surgery, but a range of intracardiac and epicardial procedures. The aim of the study was, therefore, to demonstrate the breadth of clinical applicability of the RAS approach to diverse cardiac procedures. As reported by Balkhy et al., over a 7-year period, a total of 1,103 robotic-assisted cardiac surgeries were conducted, with the majority being off-pump, totally endoscopic coronary artery bypasses (53%) [18]. Intracardiac procedures accounted for 36% of the cases, including various operations such as mitral and tricuspid valve repairs, septal defect repairs, and aortic valve replacements. Epicardial electrophysiology-related procedures, such as atrial fibrillation ablation and left atrial appendage ligation, comprised 7% of the surgeries, while the remaining 4% involved other epicardial procedures, such as pericardiectomy. The overall mortality rate was 1.2%, with fewer deaths than expected (observed-expected ratio of 0.7). Mortality was low, at 1.0% for coronary bypass and 1.5% for intracardiac procedures. Conversions to open surgery occurred in only 0.7% of cases, and 2.2% required reoperation for bleeding. Mean hospital stay was 2.74 days, with an ICU stay of 1.28 days. By demonstrating safe and efficient application across a wide age range (18–91 years; mean 59), the findings of Balhky et al. naturally raise the question of whether similar advantages could, in the future, be extended to cardiac procedures most relevant to neonates [18].

More recently, Koulaouzidis et al. extended the applicability of the RAS approach in adult cardiac care. Their study considered a broad range of applications across cardiac surgery, including Coronary Artery Bypass Grafting (CABG) and Aortic Valve Replacement (AVR). They also explored its potential in interventional cardiology, such as robotic percutaneous coronary intervention (robotic-PCI) and intracardiac shunt procedures like atrial septal defect (ASD) repair, as well as routine on-ward tasks. Regarding procedures, this study reports on a retrospective analysis of 484,128 CABG cases, including 2,582 robotic procedures, in which a robotic CABG was associated with lower rates of postoperative stroke (0.0% vs. 1.5%) and transfusion (13.5% vs. 24.4%), though mortality rates were similar between robotic and conventional methods. A multicentre Dutch trial showed shorter hospital stays for robotic CABG (5 vs. 7 days, *p* < 0.01) with similar one-month quality-of-life outcomes. In mitral valve repair, cohorts from the Cleveland Clinic (759 patients) and China (234 patients) demonstrated shorter hospitalisation (4.2 vs. 5–6 days) and fewer complications, despite longer bypass and cross-clamp times. Robotic AVR, though less common, appears feasible in patients unsuitable for sternotomy or TAVI, but prolonged operative times remain a limitation. For ASD repair, a comparative series of 508 cases reported shorter ICU and overall hospital stays with robotic techniques, with comparable safety profiles [23].

### Current state of robotic CHD surgeries in the neonatal population

**Fig. 1:**
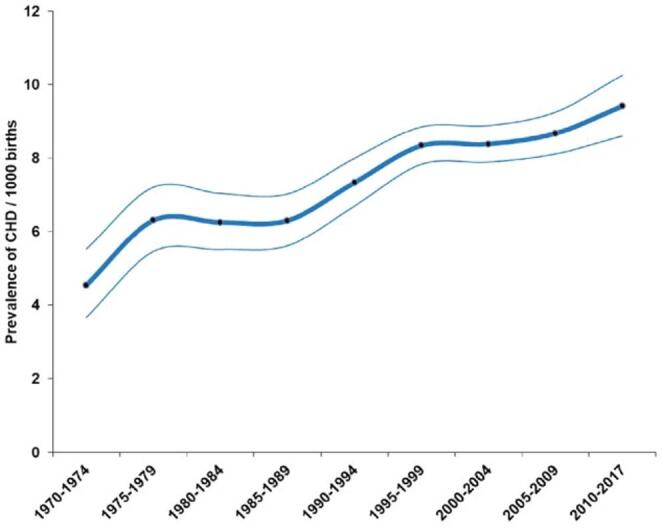
Changes in birth prevalence of CHD 1970–2017, with thick line estimating overall prevalence and thin line presenting 95% CI [16]

**Fig. 2:**
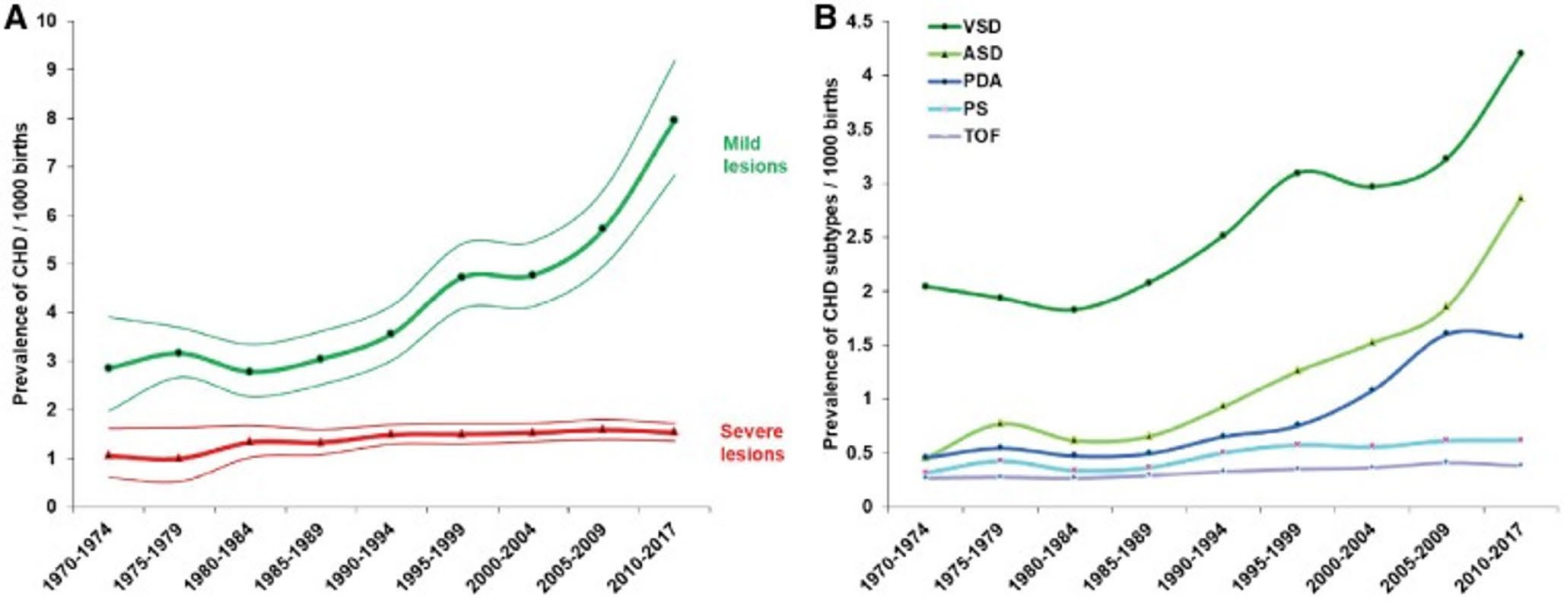
Changes in birth prevalence of CHD by type, 1970–2017; Ventricular Septal Defect (VSD), Atrial Septal Defect (ASD), Patent Ductus Arteriosus (PDA), Pulmonary Stenosis (PS), and Tetralogy of Fallot (TOF) [16]

A RAS approach to the repair of a CHD in neonates has not been described. CHD arising from structural abnormalities, are among the most common birth defects, affecting over 1% of newborns [25], and accounting for nearly one third of congenital anomalies [26]. As highlighted by Kiraly (2022), approximately 50% of these patients with CHD require cardiac surgery during their lifetime, with half of these procedures occurring within the first six months of life [27]. Among congenital heart defects, the most common is VSD, with a reported incidence of 1.5 to 3 per 1000 term infants and 4.5–7.5 per 1000 pre-term infants [28]. Another systematic review and meta-analysis of 260 studies estimated the mean prevalence of CHD from 2010 to 17 to be 9.4 per 1,000 live births [16]. Moreover, birth prevalence of CHD is increasing (see Fig. 1). Liu et al. (2019) reported that between 1970 and 2017, the global prevalence of CHD increased by 10% every 5 years, largely due to improved detection of milder lesions such as VSD, ASD, and PDA. ASD prevalence saw the highest increase (see Fig. 2), with a 6-fold rise from 1970 to 75 to 2010-17 [16]. The rising prevalence of CHD arguably underscores the urgency for further innovation in this area. Regarding neonatal cardiothoracic heart surgery, CHA most typically requiring surgical intervention include PDA, ASD, VSD, Truncus Arteriosus (TA), Total Anomalous Pulmonary Venous Return (TAPVR), Left Ventricular Outflow Tract Obstruction (LVOTO), Coarctation of Aorta (CoA), Tetralogy of Fallot (ToF), Transposition of the Great Arteries (TGA), Tricuspid Atresia (TrA), and Ebstein’s Anomaly (EA) [28]. Among these listed, several CHA lesions require timely surgical intervention during the neonatal period, without alternative. TA necessitates surgical intervention, corrected via the Rastelli procedure within the first few weeks of life [28]. Although relatively rare (with an incidence of 3 to 10 per 100,000 live births), TA accounts for 4% of all critical congenital cardiac anomalies [29], requiring early and urgent surgical intervention. TGA similarly necessitates surgical repair via the Jantene Procedure within the first two weeks of life [28]. The Jantene procedure for TGA has a notable history as a technical innovation: commonly regarded as symbolising progression and the continuous need for reappraisal and invention within cardiac surgery [30]. Surgical repair of EA should also be performed as soon as possible after birth, during the neonatal period. This is usually done via reconstruction of the tricuspid valve and closure of ASD, or the Fontan procedure [28].

## Analysis

### Evidence for the procedural applicability of a RAS approach to CHA via RAS repairs in later life

Prior work demonstrates that RAS is both feasible and beneficial for repairing CHA in older patients, supporting its procedural applicability. At the Department of Cardiovascular Surgery, Istanbul Mehmet Akif Ersoy Thoracic and Cardiovascular Surgery Hospital, a retrospective analysis of 242 patients (mean age 30.9 ± 12.1 years) who underwent robotically assisted congenital cardiac surgery with the *da Vinci Si* system (May 2013–February 2020) included cases such as ASD, PAPVC, and VSD [14]. Contraindications included prior cardiothoracic surgery and chest wall deformities. A 3 cm port enabled ASD, PAPVC, and left atrial membrane repairs, with improving bypass and clamp times across the series. Outcomes were excellent: no mortality, a 0.8% conversion rate, low complications (stroke 0.4%, cardiac 2.4%), a mean stay of 3.5 days, and 87.6% avoiding transfusion. At 3.6-year follow-up, no residual defects were reported. While demonstrating safety and efficiency, the series included no neonatal cases, highlighting the need for future study in younger patients.

### Laparoscopic and thoracoscopic approaches in neonatal cardiothoracic surgery: evaluating current tolerability and success of MIS as a prelude to RAS

**Fig. 3:**
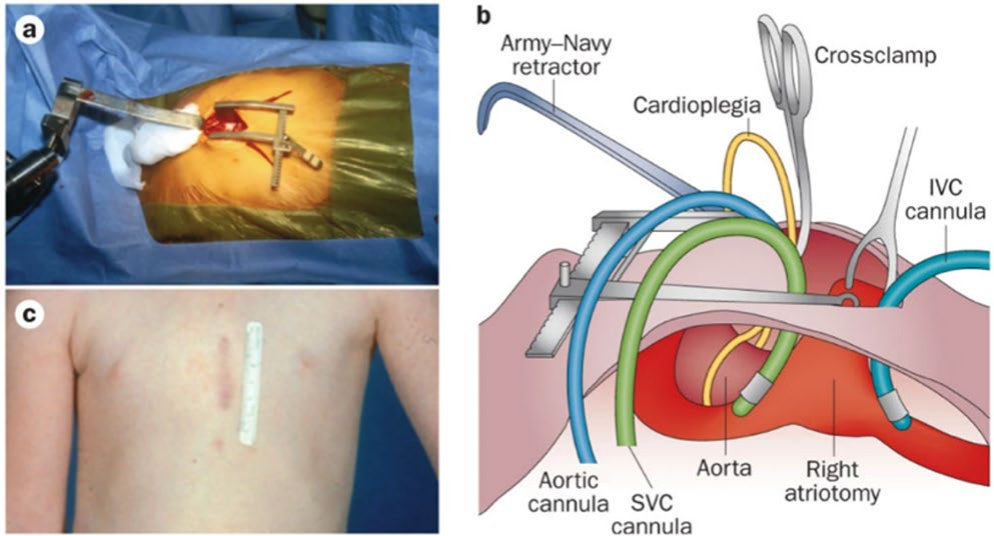
Mini Sternotomy: The technique uses a 2–3 cm midline xyphoid incision (**a**), exposing only the lower third of the sternum to maintain upper-sternal stability. A parasagittal view shows the cephalad-retracted incision with cannula and instrument placement (**b**), and panel (**c**) demonstrates a healed lower sternotomy scar after tetralogy of Fallot repair. Figure reproduced from Minimally Invasive Paediatric Cardiac Surgery, Emile Bacha et al., Nature Reviews Cardiology (2014), with permission from Springer Nature [32]

Minimally invasive surgery (MIS), including laparoscopic and thoracoscopic methods, transformed paediatric surgery by reducing the physiological stress of open operations [31]. These approaches are especially beneficial in neonatal patients, where reducing surgical stress is crucial due to their delicate physiology. Thoracoscopic surgery, applied in procedures such as the repair of congenital diaphragmatic hernia and other lung-related conditions, offers improved visualisation and precision, while minimising postoperative complications [32]. Similarly, laparoscopic techniques have proven effective in managing abdominal and thoracic conditions in neonates with congenital heart disease, showing comparable or superior outcomes to open surgery [20]. Recent evidence shows that infants with CHD can safely undergo minimally invasive techniques (MIT), even in non-cardiac surgeries, highlighting their broader potential. Figure 3 illustrates a mini-sternotomy procedure. This subsection reviews the success and tolerability of these techniques in paediatric cardiothoracic surgery, supporting further research into robotic approaches to refine and extend these benefits to neonates.

### Thoracoscopic lobectomy in paediatric surgery: benefits, challenges, and future directions

Thoracoscopic lobectomy (TL) involves resecting lung tissue to treat congenital lesions. In infants, it is a well-established, safe, and effective procedure that offers reduced pain, shorter hospital stays, and lower long-term morbidity, including decreased risk of chest wall deformity [33, 34]. Lung resections in infants and children are commonly performed for congenital lung lesions such as bronchopulmonary sequestrations, congenital lobar emphysema and congenital pulmonary airway malformation [35, 36]. While many of these lesions are detected prenatally (by ultrasound) and remain asymptomatic at birth, some present with respiratory distress later [37, 38]. Although conservative management is sometimes advocated, complete lobar resection is generally recommended. MIT offer reduced pain and morbidity, making them the preferred surgical approach [39]. The success of MIT in paediatric patients supports further investigation into advanced methods, particularly robotic approaches, which could enable complex cardiothoracic procedures in neonates in the future.

A paediatric surgical evaluation involving infants with a mean age and weight of 72 days and 4.2 kg, respectively, assessed the outcomes of thoracoscopic lobectomy for congenital lung lesions [39]. The study demonstrated that 193 out of 194 thoracoscopic procedures were successfully completed, with an average operative time of 82 min. The procedures included 50 upper, 8 middle, and 136 lower lobe resections. Intraoperative complications were minimal, occurring in only 2.1% of cases, with just one instance requiring conversion to open surgery due to bleeding. Postoperative complications were similarly low, with a 3.1% rate and only one patient requiring re-exploration for a prolonged air leak. The average hospital stay was 1.8 days, with no conversions to open surgery or blood transfusions in the past 15 years [39]. These positive outcomes highlight the effectiveness of MIT in paediatric surgery and underscore the potential for further advancements in surgical methods, such as a RAS approach. Similarly, additional insights into neonatal MIS, including both laparoscopy and thoracoscopy, were highlighted in a study of 49 neonates undergoing 50 procedures (mean age 11 days, weight 3285 g) [40]. Operative time averaged 79 min. Oxygen saturation fell during thoracic insufflation or high-pressure pneumoperitoneum, with temporary pauses required in 6% of cases. Systolic pressure decreased in 20% of neonates but responded to fluid expansion, and postoperative heat loss correlated with insufflation duration [40]. The absence of complications highlights MIS feasibility in neonates with proper precautions. These results stress the need for precise planning and technique, potentially enhanced by future robotic-assisted approaches.

Although thoracoscopic lobectomy offers clear benefits, its adoption remains limited due to technical complexity and the infrequency of such cases [41]. Managing large pulmonary vessels and operating without tactile feedback add to the difficulty. In neonates, the small operative field and proximity to vital structures increase the challenge. A case involving a 25-day-old neonate undergoing thoracoscopic excision of a bronchogenic cyst illustrates both the potential and risks: an iatrogenic left bronchus injury occurred but was successfully repaired thoracoscopically, avoiding conversion to open surgery. This case demonstrates the steep learning curve and technical precision required for neonatal thoracoscopy, as well as its potential for safely managing complex complications [42]. These cases underscore the need to refine current techniques, with robotic systems offering a potential means of overcoming existing limitations. Early thoracoscopic lobectomy provides faster recovery, fewer complications, and better outcomes in experienced centres, despite technical demands. Further technological advancement, including robotic integration, may enhance precision and help extend these benefits to neonatal cardiothoracic surgery.

### Tolerability of laparoscopic procedures in CHD patients and implications for advancing paediatric cardiothoracic surgery

Building on the success of thoracoscopic techniques, both thoracoscopic and laparoscopic surgery has also shown promise in managing complex paediatric conditions, including in those neonates with CHD [20, 43, 44]. Bergmeier and Schier evaluated thoracoscopic surgery in 15 neonates with cardiac anomalies undergoing oesophageal atresia or diaphragmatic hernia repair. The median operative time was 177 min, with intrapleural pressure of 9 mm Hg. Despite complex anomalies such as tetralogy of Fallot and septal defects, only one patient developed haemodynamic instability requiring short-term dobutamine. These results demonstrate the feasibility of thoracoscopic surgery in neonates with cardiac defects, indicating such anomalies need not preclude minimally invasive approaches [45]. A comparison of laparoscopic and open surgical procedures in 111 children with CHD revealed that laparoscopic surgery is both safe and effective, even in this high-risk population [43]. Conducted over a ten-year period, the study involved 121 laparoscopic procedures, primarily gastrostomy tube placements and fundoplications, with a median patient age of 2.5 months. The laparoscopic group experienced a low conversion rate to open surgery (6.6%) and a minor complication rate of 5%, including issues such as postoperative bleeding and surgical site infections. Importantly, no intraoperative haemodynamic instability occurred, and all patients survived 30 days post-surgery. The open group, though similar in operative time, had more complications, including two cardiac-related deaths. Outcomes were comparable between single- and double-ventricle physiology, reinforcing the safety of laparoscopic techniques in complex CHD. These findings challenge the traditional preference for open surgery, showing laparoscopic approaches reduce recovery time and complications, while highlighting the potential for robotic innovation to further improve paediatric cardiothoracic outcomes.

A large national study from the United States evaluating the safety and effectiveness of laparoscopic surgery (LS) in infants with CHD provides further compelling evidence supporting the use of MIT in this high-risk population [20]. The study, which included 3684 infants, compared the outcomes of 1182 laparoscopic procedures to 2502 open surgeries. The results demonstrated that laparoscopic surgery is associated with significantly better outcomes, including a lower overall complication rate (15.7% vs. 36.4%, *P* < 0.001) and a shorter median hospital stay (7 days vs. 15 days, *P* < 0.001). Additionally, laparoscopic surgery led to fewer specific complications, including pulmonary complications (5.5% vs. 10.4%, *P* < 0.001) and bleeding (7.0% vs. 25.3%, *P* < 0.001). Notwithstanding the complex physiological challenges associated with CHD, the study found no significant difference in 30-day mortality between laparoscopic and open surgery (1.4% vs. 4.8%, *P* = 0.28), further supporting the safety and efficacy of laparoscopic techniques in this vulnerable patient group. However, the study also noted a higher 30-day readmission rate for laparoscopic procedures (12.4% vs. 5.6%, *P* < 0.001), suggesting that while LS is safer overall, there are areas for improvement. Lower complication rates and comparable mortality highlight the potential of MIT to improve outcomes. These results support wider adoption of laparoscopic methods and suggest that robotic surgery could further enhance precision and safety in paediatric cardiothoracic procedures.

Additionally, a large-scale study comparing outcomes of laparoscopic and open surgeries in children with CHD provides significant evidence supporting the use of laparoscopic surgery in this high-risk population [44]. The study involved 45,012 matched pairs of children undergoing intra-abdominal surgery, with a range of CHD severities. The results showed that laparoscopic surgery was associated with significantly lower 30-day mortality rates in children with minor CHD (0.34 [95% CI 0.15–0.79]) compared to open surgery. The in-hospital mortality and 30-day morbidity were also reduced in this group, demonstrating a consistent benefit of the laparoscopic approach. With increasing CHD severity, benefits lessened but remained, including shorter hospital stays across all groups. In severe cases, laparoscopic surgery reduced transfusions, morbidity, and surgical site infections. Higher readmission rates were observed, yet overall advantages—lower mortality and morbidity—support its value and the potential for robotic integration to further improve precision and outcomes in paediatric cardiothoracic surgery.

The studies on laparoscopic surgery in children with CHD collectively reveal a range of challenges that underscore the complexity of applying MIT in this high-risk population. The first study highlighted the risks associated with increased intra-abdominal pressure and CO2-induced hypercapnia, which can lead to decreased cardiac output and hypoxemia. In this context, 6.6% of cases required conversion to open surgery due to technical difficulties, such as dense adhesions and inadequate stomach size, with a 5% complication rate that included postoperative bleeding and wound infections [43]. A second national U.S. study of 34,543 laparoscopic cases found that in children with minor CHD (*n* = 1349), mortality was comparable to those without CHD, but morbidity was significantly higher (OR 1.71, 95% CI 1.37–2.13). In contrast, both morbidity and mortality rose markedly in children with major CHD (*n* = 1106; morbidity OR 2.07, mortality OR 3.46) and severe CHD (*n* = 266; morbidity OR 2.51, mortality OR 12.31). These findings indicate that, whilst laparoscopy offers clear benefits, significant risks persist in this patient group [46].

The third study added that while laparoscopic surgery showed advantages like shorter hospital stays and reduced transfusion needs, these benefits were less pronounced in patients with severe CHD. Specifically, the study found that lower 30-day mortality (0.34 [95% CI 0.15–0.79]) and morbidity rates were most significant in children with minor CHD, while those with severe CHD experienced limited gains and faced increased unplanned readmissions [44]. Given these challenges, robotic surgery presents a promising solution. With superior precision, control, and visualisation, it may reduce haemodynamic instability, open conversions, and complication rates. This advancement could enhance safety and effectiveness in paediatric CHD surgery and extend MIS benefits to more cardiothoracic cases, marking an important step toward safer, more effective neonatal care, especially for severe CHD.

### Robotic surgery in neonatal and other paediatric specialties

Having highlighted the benefits of robotic surgery in adult cardiothoracic procedures and advancements in minimally invasive paediatric approaches, this section explores the application of robotic-assisted surgery (RAS) in paediatric and neonatal populations across other systems. RAS has shown optimal outcomes in adult procedures, such as prostatectomies and mitral valve repairs, due to enhanced precision, fewer complications, and faster recovery, driving its adoption in paediatric surgeries. Platforms like the Da Vinci Surgical System offer advantages such as improved control, reduced tremor, and superior 3D imaging, particularly valuable in neonatal and paediatric surgeries where precision in confined spaces is critical for better outcomes [47, 48]. The following subsections explore the outcomes of RAS approaches in four key paediatric and neonatal surgeries—mediastinal mass excision, lobectomies, CDH repair and OA repair—while highlighting the specific advantages and challenges associated with this innovative surgical approach.

### Robotic-assisted pulmonary resection in paediatric surgery

In paediatric patients, Lobectomy is commonly indicated for conditions such as congenital pulmonary airway malformation (CPAM), intralobar pulmonary sequestration (IPS), and severe bronchiectasis. Traditionally, lobectomies were performed via open thoracotomy, requiring a large chest incision, which allowed excellent visualisation but resulted in significant postoperative pain and longer recovery times. Over time, VATS became the gold standard for many thoracic procedures, offering a less invasive approach with smaller incisions, reduced trauma, and faster recovery. However, in more complex cases, especially involving paediatric patients, VATS can present challenges due to the confined spaces and delicate structures involved. These complexities highlight the suitability of RAS for lobectomy, which provides enhanced precision and control in such environments. Recently, Liang et al. (2024) conducted a retrospective cohort study comparing RAS and VATS in paediatric patients with pulmonary sequestration [49]. The study included 170 patients—93 undergoing RATS and 77 VATS. Median age and weight were 10 months (IQR 7–25) and 9.5 kg (IQR 8.5–13.1) for RATS, and 9.5 months (IQR 7–17.5) and 9.2 kg (IQR 8.3–12.0) for VATS. Chest tube use was significantly lower with RATS (61.3% vs. 90.9%, *p* < 0.001), and drainage duration was shorter (median 1.0 day vs. 2.0 days, *p* < 0.001). Hospital stay was also reduced (5.0 vs. 6.0 days, *p* < 0.001). Each group had one conversion to thoracotomy. Overall, RATS was deemed safe and effective for pulmonary sequestration in children > 6 months and > 7 kg, with better short-term outcomes than VATS. A study by another group corroborates these findings in a retrospective comparative study between RAS and VATS in paediatric lobectomies [50]. The study included 74 paediatric patients undergoing pulmonary resections via RAS (*n* = 29) or VATS (*n* = 42), with comparable age and weight profiles; the youngest RAS patient was 6 months old and 8 kg. There was one conversion to thoracotomy in the RAS group (pulmonary vein bleeding) and two in the VATS group (inflammatory adhesions). Total operative time was longer with RAS (148.3 ± 36.8 vs. 118.3 ± 22.5 min, *p* < 0.001), though pure operative time favoured RAS (103.9 ± 28.5 vs. 111.4 ± 18.3 min, *p* = 0.045). Postoperative fever was less frequent with RAS (2/29 vs. 11/42, *p* = 0.039). Chest drainage duration, hospital stay, and complication rates were similar. Overall, RAS was deemed safe and effective for children > 8 kg, offering improved precision despite greater procedural complexity.

In cases of higher anatomical or pathological complexity, such as severe bronchiectasis, RAS has also proven beneficial. Durand et al. (2021) evaluated paediatric lobectomy outcomes for bronchiectasis and reported that RAS provided excellent visualisation and precision, even in highly inflamed and challenging areas [51]. No conversions to open surgery were necessary in the RAS group, whereas five conversions were required in the thoracoscopic group. Although the RAS group’s operation time was longer (mean 247 ± 50 min vs. 152 ± 57 min for thoracoscopy, *p* = 0.008), the reduced need for blood transfusions and fewer severe postoperative complications suggest that RAS is a safer option for complex paediatric cases [51]. Navarrete-Arellano et al. (2020) assessed RAS in paediatric patients, including lobectomies, diaphragmatic plications, and bronchogenic cyst resections. In this study RAS demonstrated a 20% conversion rate and a 10% complication rate, highlighting its feasibility and safety as a minimally invasive alternative. The study emphasised the benefits of superior visualisation and precision in complex cases while noting the importance of expertise and patient selection to optimise outcomes [52]. While longer operative times and higher initial costs are challenges, studies suggest these are outweighed by improved precision, fewer complications, and faster recovery.

### Robotic-assisted mediastinal mass excision (MME) in paediatric surgery

Similar to its success in paediatric lobectomies, MME has also benefited from the precision and control of robotic-assisted surgery. MME involves removing abnormal mediastinal growths—such as neurogenic tumours, thymic tumours, and congenital cysts—that can compress nearby structures and cause respiratory or cardiovascular issues. The mediastinum’s complex anatomy and confined space pose significant challenges, especially in paediatric patients with small, delicate structures. Technical demands include careful dissection around vital organs, limited exposure, and complete mass removal without injury. When lesions are adherent to major vessels or the trachea, meticulous technique and planning are essential to prevent haemorrhage, airway compromise, or nerve damage. This procedure has already been shown to benefit from MIS techniques with better visualisation, decreased trauma, blood loss and length of hospital stay [53, 54] and may further benefit from RAS intervention. Ballouhey et al. (2015) demonstrated the benefits of robotic-assisted thoracic surgery (RATS) for paediatric mediastinal cyst excisions. Their study reported a hospital stay of 4.2 days for RATS, which was shorter compared to 6.6 days for thoracotomy and within the range observed for thoracoscopy (2.6–6 days). Additionally, RATS achieved a 0% conversion rate, contrasting with conversion rates of 18–20% for thoracoscopy. Although RATS involved longer operative times (146 min) compared to thoracoscopy (78–99 min), it remained competitive with thoracotomy (115 min in one study). These findings highlight the precision and safety of RATS for mediastinal cyst excisions, particularly in children over 20 kg, while underscoring the need for technological improvements to better accommodate smaller patients [55].

More recently, Svetanoff et al. (2023) reviewed the use of RAS for thoracic and mediastinal tumour resections in paediatric patients [56]. They highlighted individual cases such as those described by Meehan et al. (2008), where four patients aged 2 to 17 years underwent successful RAS (mediastinal mass resection) procedures with an average operative time of 115 min, no conversions to open surgery, and no complications such as tumour spillage and with no recurrence in any patient within 2 years [57]. Another case, Nemoto et al. (2022), reported the successful robotic-assisted resection of a ganglioneuroma in a 15-year-old female, which preserved critical structures, including the artery of Adamkiewicz. The patient experienced an uneventful postoperative course, with no recurrence observed at the 18-month follow-up. The study highlighted the advantages of RAS, such as enhanced three-dimensional visualisation and precise articulation in confined spaces, enabling safe and effective removal of tumours in anatomically challenging locations [58]. These findings underscore the potential of RAS in MME for paediatric patients, offering enhanced precision and reduced complications. However, technical challenges and the need for further refinement in technology and training remain barriers to its widespread adoption.

### Robotic-assisted congenital diaphragmatic hernia repair in neonates

While RAS is becoming more common in paediatric thoracic procedures, its use in neonatal surgeries remains relatively rare due to technical challenges. However, a case study by Meehan and Sandler (2007) demonstrated that a RAS approach is feasible even in neonates with congenital defects requiring complex interventions [59]. They reported the successful robotic repair of a Bochdalek congenital diaphragmatic hernia (CDH) in a 2.2-kg neonate using the Da Vinci system. CDH involves herniation of abdominal organs into the thorax, most often through a posterolateral Bochdalek defect, and requires reduction of the viscera and closure of the diaphragmatic gap. Meehan and Sandler showed that robotic articulation improves visibility and allows accurate posterolateral suturing that is challenging with standard laparoscopy. Although instrument movement was limited and visceral reduction difficult, the 3-hour procedure was completed without complications or recurrence, supporting feasibility while noting that conventional tools may still be required in the smallest patients [59]. Further evidence from Ballouhey et al. (2014) and Navarrete-Arellano et al. (2020) supports the feasibility of RAS for congenital diaphragmatic hernia (CDH) repair in larger paediatric patients. Ballouhey et al. reported two cases of CDH repair, with an average operation time of 190 min and no complications or recurrences observed during a follow-up of 26.9 months. They noted that while RAS demonstrated benefits such as precise suturing, its feasibility was significantly reduced in smaller patients due to limited operative space and challenges with trocar placement, often leading to increased preparation time or conversion to open surgery [55]. Similarly, Navarrete-Arellano et al. reported successful robotic-assisted diaphragmatic repairs in larger paediatric patients, noting short postoperative recovery and clear technical advantages. However, they emphasised that 8-mm instruments are difficult to manoeuvre in small thoracic cavities and that meaningful neonatal application will require instrument miniaturisation and careful operative planning [52].

### Robotic-assisted Oesophageal Atresia repair in neonates

**Fig. 4:**
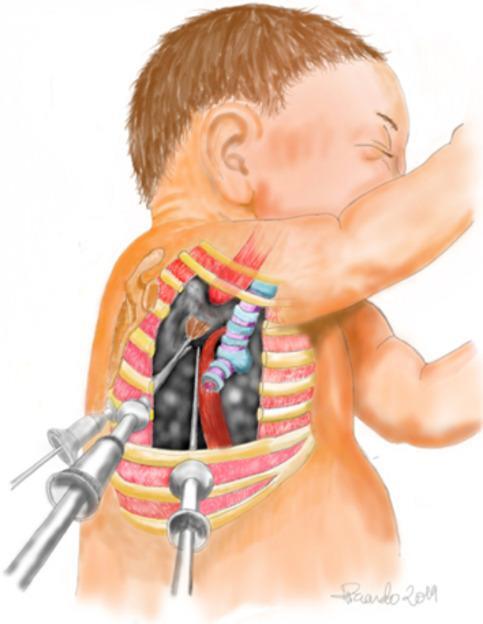
VATS Oesophageal Atresia Schematic [62]

Oesophageal atresia (OA), shown in Fig. 4, is a congenital anomaly where the oesophagus is incompletely formed, leaving its upper and lower segments disconnected. This condition, often accompanied by a tracheoesophageal fistula (TEF), requires surgical correction shortly after birth to enable feeding and proper respiratory function. TEF further complicates OA by creating an abnormal connection between the oesophagus and trachea, leading to respiratory risks. Standard OA repair involves resecting the fistula (if present) and anastomosing the oesophageal ends to restore continuity. While traditional open and minimally invasive techniques are effective, they are challenging in neonates due to the small operative field and delicate tissues. RAS offers enhanced precision and control, making it a promising option for this complex procedure.

Ferrero et al. (2022) examined robotic-assisted thoracoscopic surgery (RATS) for oesophageal conditions in paediatric patients through a retrospective multicentre study. Of 18 procedures performed, four involved OA, with a conversion rate of 11% due to exposure difficulties in neonates. The study highlighted significant limitations, particularly the incompatibility of trocar size with the small intercostal spaces of low-weight children, notably those under 15 kg. In spite of these challenges, RATS was successful in several cases and demonstrated technical advantages, such as enhanced precision and reduced postoperative complications, for children above the 15-kg threshold. Ferrero et al. concluded that while RATS holds promise, further innovations, including miniaturisation of instruments, are essential for broader application in neonates [60]. A Similarly, Li et al. (2021) reported two neonatal robotic OA repairs (3.1 kg and 3.3 kg) using asymmetric port placement and stepwise trocar insertion to overcome thoracic space constraints. Operative times were 90–95 min, with anastomoses completed in 27–30 min. One patient recovered uneventfully, while the other developed aspiration pneumonia and later a recurrent fistula requiring thoracotomy. The authors concluded that although RAS enhances precision and control in delicate anastomoses, its long-term safety in neonatal OA repair warrants further evaluation [61]. Both Ballouhey et al. (2014) and Navarrete-Arellano et al. (2020) included OA repair among the procedures performed using robotic-assisted surgery in their paediatric thoracic series. Ballouhey et al. reported three cases of OA, with a mean operative time of 195 min. Two of these cases required conversion to open surgery due to space constraints in neonates weighing approximately 3 kg. They highlighted that while RAS facilitated precise anastomoses, the large size of the 8-mm instruments and limited visibility posed significant challenges in smaller patients. Similarly, and as mentioned earlier, Navarrete-Arellano et al. (2020) noted that the restricted availability of instrument sizes, with robotic tools limited to 8 mm and 5 mm, compounded the technical difficulties in neonates, underscoring the importance of further advancements in robotic technology to improve its feasibility in smaller children​ [52, 55].

RAS has demonstrated success in low-weight neonates, showing that, with precise techniques and careful planning, complex procedures like OA repair can be effectively managed even in patients as small as 3 kg [50]. However, consistent pattern of limitations among all the studies such as the size of robotic instruments, restricted visibility, and the difficulty of operating in confined thoracic spaces present ongoing challenges, particularly in infants under 5 kg. These issues often lead to a need for conversion to open surgery. To make RAS more widely applicable in neonatal cases, advancements in instrument miniaturisation and improved surgical protocols are crucial. Addressing these limitations could significantly enhance the safety and effectiveness of robotic surgery in this delicate patient population [41, 44].

## Anatomical considerations for cardiothoracic procedures in neonates with congenital heart disease and robotic adaptations

### Anatomical considerations for neonatal intracardiac procedures

In neonatal cardiothoracic surgery, the most common CHA requiring surgical intervention include Patent Ductus Arteriosus (PDA), Atrial Septal Defect (ASD), Ventricular Septal Defect (VSD), Tricuspid Atresia (TA), Total Anomalous Pulmonary Venous Return (TAPVR), Left Ventricular Outflow Tract Obstruction (LVOTO), Coarctation of the Aorta (CoA), Tetralogy of Fallot (ToF), Transposition of the Great Arteries (TGA), Truncus Arteriosus (TrA), Ebstein’s Anomaly (EA) [28]. Additional critical conditions that often require surgery to restore proper circulation include Hypoplastic Left Heart Syndrome (HLHS), where the left side of the heart is underdeveloped, and Pulmonary Atresia (PA), characterised by an absent pulmonary valve. Interrupted Aortic Arch (IAA), where the aorta is incomplete, Double Outlet Right Ventricle (DORV), and Atrioventricular Septal Defect (AVSD), which involves a central heart hole. These defects represent the major congenital heart diseases treated through neonatal cardiothoracic surgery. Across CHD, there are several general anatomical challenges inherent to most neonatal heart surgeries, including the small size of cardiac structures, the fragility of neonatal tissues, and the limited surgical space available during procedures, regardless of the specific congenital heart defect being addressed. For the purposes of exploring specific geometric and ergonomic challenges that each of these CHA presents in neonatal cardiothoracic surgeries, these anomalies will be further classified as follows: Septal Defects (ASD, VSD, AVSD), Outflow Tract Obstructions (LVOTO, CoA), Valve Abnormalities (TrA, EA, PA), Complex Congenital Heart Defects (ToF, TGA, DORV), Vascular Abnormalities (TAPVR, IAA) and Single Ventricle Anomalies (HLHS). This paper will focus on anatomical challenges posed by septal defects with predominance of VSD and outflow tract obstructions with predominance of CoA, owing to their prevalence amid CHD.

Among Septal Defects, VSDs are the most common form in children, comprising 20–25% of congenital heart disease [63]. Subsequently, it follows that the most common septal defect requiring surgical repair in neonates today is VSD. There are currently three main surgical approaches for VSD repair: the traditional on-pump method, the interventional approach, and the hybrid method, which is a minimally invasive periventricular device closure performed off-pump. Of the aforementioned, the traditional on-pump method is considered “gold standard” [64]. Prognosis has been linked to anatomical factors, including location and size of the defect. A key anatomical challenge for neonatal VSD repair includes relatively small size of VSD, requiring precise suturing. Complications may arise from this anatomical constraint resulting in residual VSD, defined as significant residual VSD greater than 3 ml post-operatively [65]. Whilst the challenge posed by small size, some studies nevertheless recommend performing VSD surgery as soon as possible [66]. A retrospective study by Mirzaaghayan reviewed 54 patients undergoing VSD closure surgery between March 2014 and 2017 with a mean weight of 4.4 ± 0.61 kg, concluding that timely surgical VSD closure is recommended on the basis that earlier surgery prevented further complications, such as heart failure and failure to thrive, making early closure beneficial for patient outcomes [66]. Given these opposing challenges, a RAS approach might theoretically help in resolving the dilemma between the need for early intervention and the difficulty in achieving precision.

Of the Outflow Tract Obstructions, CoA is most common among the neonatal population, requiring surgical intervention in early life. CoA is a prevalent congenital heart defect, affecting an estimated 4 in every 10,000 live births [67]. The treatment for coarctation of the aorta involves removing the narrowed section of the vessel. This can be achieved through surgical methods or transcatheter techniques [68]. In surgery, the constricted segment is excised, followed by anastomosis of the healthy sections of the aorta [68]. Anatomical challenges during CoA surgery stem from the narrow and underdeveloped aortic arch in neonates, particularly at the aortic isthmus, posing difficulties in ensuring adequate resection and anastomosis. In neonates, the risk of recoarctation following surgical intervention is approximately 10% [68]. A study by Burch et al. (2009) reviewed 167 neonates and infants under 90 days old who underwent CoA repair between 1996 and 2006 and assessed the recurrence of coarctation and survival rates, with special attention to low-weight infants (under 2.5 kg) and their risk of needing reintervention [69]. This study described the anatomical challenges specific to neonatal CoA repair and identified that re-coarctation in neonates often occurs due to factors like small transverse arch diameter, arch anatomy, and improper suture material – polypropylene sutures were linked to higher recurrence rates compared to polydioxanone [69]. RAS has not yet been applied to neonatal CoA repair, but its precision and dexterity could help manage small arch diameters, ensure full ductal tissue removal, and reduce re-coarctation from poor suture placement. Current systems, however, are not suited to patients under 5 kg, limiting these potential benefits.

### Introduction to current adaptations

Surgeons have adapted existing RAS systems, such as the Da Vinci platform, to meet the challenges of neonatal and paediatric surgery. Key modifications include altered port placements to fit limited intercostal spaces, often using asymmetric configurations to reduce robotic arm collisions and ensure adequate reach [70]. Stepwise trocar insertion, involving gradual dilation, is another vital adaptation to prevent tissue injury—especially in procedures like oesophageal atresia repair, where preserving small structures is critical [60]. The transition to using 5 mm instruments, such as the 5 mm EndoWrist Needle Driver and the 5 mm Maryland Bipolar Forceps, has been a significant advancement in paediatric robotic surgery. These smaller instruments offer improved manoeuvrability in confined spaces but have limitations in dexterity and range of motion compared to their 8 mm counterparts [71]. The Versius system, as described by Brownlee and Slack, helps overcome several limitations by using modular robotic arms and wristed instruments that pass through 5-mm ports, improving flexibility and precision in small operative fields. Its modular design allows tailored arm configurations and optimised port placement, reducing external collisions in narrow paediatric thoraces and improving instrument handling. The 2.71-cm needle driver suits small spaces but still offers limited articulation for complex manoeuvres [71]. The absence of curved 5 mm instruments, essential for delicate dissections in confined spaces, further limits the ability to perform certain intricate procedures, such as precise vascular anastomoses [72] which is likely to be a necessity in neonatal cardiothoracic surgery.

However, the Versius system’s incorporation of enhanced articulation capabilities through its unique wristed instruments overcomes some of these challenges, facilitating more precise manoeuvres and reducing the risk of tissue damage during complex dissections and suturing in paediatric patients [73]. Furthermore, ergonomic modifications, including patient positioning strategies, are crucial for optimising access to vital anatomical structures. For example, using foam padding to elevate the patient from the operating table increases the external range of motion for robotic arms, reducing the risk of collisions between the robotic system and the table. This technique also provides more space for the bedside assistant, which is essential in small paediatric and neonatal patients [72]. Specific positioning methods, such as placing the patient in a lateral decubitus position with a “bump” under the affected side, have been employed to improve access during thoracic surgeries, enabling better visualisation and safer instrument navigation [72]. Additionally, the implementation of tilt and rotation adjustments of the operating table during procedures has been shown to enhance surgical access and visibility. For example, the use of Trendelenburg or reverse Trendelenburg positions, depending on the location of the surgical target, facilitates easier instrument manoeuvring and more effective use of robotic capabilities [73]. These positioning strategies are vital not only for improving surgical precision but also for ensuring patient safety by preventing accidental injuries caused by robotic arm movements. Even with these adaptations, major challenges persist, particularly due to the limited dexterity and flexibility of current robotic instruments.

## Towards feasibility: anatomical and technological steps needed for neonatal robotic cardiac surgery

### Anatomical and technological barriers preventing current robotic systems from functioning in neonates

Despite the introduction of many adaptations, significant challenges remain, particularly regarding the limitations of current robotic instruments in terms of dexterity, flexibility, and visualisation. For instance, while 5 mm instruments provide better access in small spaces, they have reduced range of motion and articulation compared to their 8 mm counterparts, requiring more space for full articulation. This limitation can hinder the performance of complex manoeuvres in confined spaces, such as those required for precise vascular anastomoses in neonatal cardiothoracic surgery [71]. Additionally, the lack of curved 5 mm instruments, which are critical for delicate dissections, limits the ability to perform intricate procedures, particularly in the small and complex spaces found in neonates [72]. Visualisation constraints are another major challenge. The original 12 mm 3D scope used in the Da Vinci system is unsuitable for neonates, leading to the adoption of a 5 mm 2D scope that, while improving access, lacks the necessary 3D visualisation crucial for precision in complex procedures. The introduction of the 8.5 mm 3D scope offers better visualisation but is still too large for neonates under 5 kg, limiting its application [71]. Maintaining clear visualisation in the small operative fields of neonates is made more complex by the rigidity of the thoracic cavity and the difficulty in sustaining pneumoperitoneum, highlighting a gap in the current system’s adaptability to neonatal anatomical constraints [49]. Additionally, haptic feedback is another critical limitation in current systems. The inability to feel resistance can lead to inadvertent tissue damage, especially in the delicate anatomy of neonates. Advanced sensors, have the potential to address this issue by providing real-time feedback on force and tactile sensation, enabling surgeons to better gauge the resistance of tissues during procedures. High conversion rates to open surgery remain a concern, with procedures like robotic-assisted thoracoscopic surgery (RATS) reporting conversion rates as high as 61.5%. These conversions are often due to inadequate space for instrument manoeuvring and challenges in delineating neonatal anatomy [70]. Such conversion rates highlight the need for more versatile robotic systems that can adapt to the unique anatomical limitations of neonates.

### Experimental barriers to developing neonatal robotic cardiothoracic surgery

Despite advances in robotic cardiac surgery simulation, there remains no neonatal-specific benchtop or animal model capable of supporting iterative instrument testing or pre-clinical feasibility work. Current robotic cardiac simulation platforms rely exclusively on adult large-animal wet-lab models, as demonstrated in the international multicentre study by Atroshchenko et al., which used adult porcine hearts (35–60 kg) and adult porcine chest walls to simulate intracardiac suturing, mitral annular stitching, and internal thoracic artery dissection. These adult-scale structures differ substantially from neonatal thoracic and intracardiac geometry. Because no validated high-fidelity neonatal model—animal or benchtop—is available, contemporary simulation cannot replicate the spatial, geometric, or physiological constraints that define neonatal cardiothoracic surgery, thereby preventing realistic robotic instrument miniaturisation or procedural testing [74]. The 2024 review by Davidson et al. highlights that foetal and neonatal surgical training still relies almost entirely on large-animal models, predominantly swine and canine, which have been the mainstay since Halsted’s introduction of animal labs in 1889. Although these models offer physiological realism, the authors emphasise that they do not replicate neonatal anatomy, particularly the extremely small operative fields, fragile tissues, and limited thoracic space. Non-animal simulation platforms—bench models, VR/AR systems, and 3-D printing—are described as useful adjuncts but remain insufficient due to limited fidelity, lack of physiological responses, and the inability to simulate neonatal tissue characteristics. Crucially, Davidson et al. state that no validated high-fidelity neonatal thoracic or intracardiac simulation models exist, meaning there is currently no platform suitable for testing robotic instrument miniaturisation, procedural rehearsal, or pre-clinical feasibility. This gap directly prevents any meaningful experimental pathway toward neonatal robotic cardiothoracic surgery [75]. Moreover, paediatric robotic downscaling has been tested using 3 mm instruments in piglets weighing a median of 6.9 kg, reflecting infant—not neonatal—anatomy. Krebs et al. showed that although abdominal and thoracoscopic procedures were feasible, key issues persisted, including excessive instrument bending, fulcrum-point recalibration, and a restricted thoracic workspace. Even in these larger models, the system operated at the limits of its mechanical tolerance. Importantly, the study offers no pathway toward a < 5 kg neonatal model, providing no evidence that current robotic systems can accommodate true neonatal thoracic geometry or support a validated neonatal-specific platform [76]. Without a neonatal-appropriate model, designers cannot test or optimise < 5 kg instrument geometry, surgical ergonomics or cardiopulmonary interactions in the confined neonatal thorax. This gap effectively halts any meaningful ‘miniaturisation-through-iteration’ pathway at present, but it also highlights a clear and addressable engineering frontier—one that, if bridged through dedicated neonatal-specific modelling and instrument development, could enable future feasibility and open a realistic translational pathway toward robotic neonatal cardiothoracic surgery.

### Lack of instrument miniaturisation research programmes

Despite the theoretical appeal of robotic approaches, there remains no dedicated instrument-miniaturisation research programme focused on neonates (< 5 kg). In current paediatric robotic systems, “paediatric” instruments remain sized at 5 mm or greater, which are still proportionally oversized for neonatal thoracic geometry and small inter-port spacing. Studies show that while 5 mm instruments may improve port access in children, they suffer from reduced articulation and require more space than 8 mm counterparts—both unfavourable in neonates [77]. Recent paediatric RAS reviews confirm that no engineering or industry pipeline targets neonatal-scale instruments. Current “paediatric” tools remain at 5 mm, still too large for neonatal thoracic geometry. As noted by Boscarelli et al., a key barrier is the difficulty in developing instruments appropriately sized for smaller children and neonates. With no platform in development for patients < 5 kg, instrument miniaturisation has stalled, leaving neonatal cardiothoracic application technologically unsupported at present, but this absence also defines a clear and tractable innovation gap—one in which targeted engineering efforts could catalyse the development of true neonatal-scale robotic tools and ultimately make future application feasible [8].

### Cardiopulmonary and ethical barriers to neonatal robotic cardiac surgery

There is no robust data assessing how neonates tolerate robotic-surgery conditions—specifically the effects of insufflation, CO₂ load and prolonged anaesthesia—during intracardiac or thoracic manipulation. Studies of neonatal laparoscopic CO₂ pneumoperitoneum indicate significant circulatory and respiratory stress in this population [78]. Without neonatal-specific experimental models, the physiological tolerance of robotic conditions—including haemodynamic stability during docking and intracardiac manipulation—remains unknown. Ethical constraints further restrict high-risk first-in-human trials, and institutional review boards cannot approve neonatal robotic cardiac surgery without a pre-clinical evidence base. These factors presently prevent early clinical translation, but they also outline a clear research agenda in developing neonatal-appropriate models and physiological data to enable future pathways toward safe robotic neonatal cardiothoracic intervention.

### Absence of robotic congenital cardiac surgery in STS data

Public reports from the Society of Thoracic Surgeons (STS) Congenital Heart Surgery Database (CHSD) (the largest congenital and paediatric cardiac surgical registry worldwide and one that captures nearly all paediatric cardiac operations performed in the United States) demonstrate detailed diagnostic and procedural classification, encompassing nearly 200 individual diagnoses and 235 distinct therapeutic interventions. In the published aggregate outcome tables, the benchmark congenital operations listed (including ventricular septal defect repair, tetralogy of Fallot repair, complete atrioventricular canal repair, arterial switch procedures, Glenn/hemi-Fontan, Fontan, truncus arteriosus repair, and the Norwood procedure) represent standard congenital cardiac surgeries, and no benchmark operation category is labelled as robotic. Given that STS CHSD captures almost the entirety of contemporary paediatric cardiac surgical activity, the absence of any robotic category in these benchmark reports indicates that robotic congenital cardiac surgery does not feature in current national reporting for neonatal or infant procedures. Separate published case reports and small series further show that robotic congenital cardiac surgery has only been applied in older paediatric or adolescent patients, typically for simple lesions such as atrial septal defect repair, rather than in neonatal or infant practice [79, 80]. Taken together, these findings confirm a clear evidence gap and underscore the need for focused research to define the developmental steps required for future neonatal applicability.

### Future innovations and directions

The development of new tools, such as the SmartArm system, represents a significant step forward in addressing many challenges. The SmartArm system, as described by Marinho et al. (2019), features two robotic arms with nine active degrees of freedom and has been validated in complex procedures such as dura mater suturing at the skull base [21]. This system’s advanced control algorithms and high degrees of freedom allow for precise movements in constrained environments, making it well-suited for the intricate and narrow operative fields typical of neonatal surgery. The incorporation of such systems might significantly reduce conversion rates and improve surgical precision. Advancements in haptic feedback and AI-driven assistance are also poised to transform neonatal robotic surgery. For example, FUTEK’s haptic feedback sensors, address issues like extraneous load compensation and temperature variations, which could enhance intraoperative control and reduce the risk of tissue damage [81]. AI algorithms might autonomously position cameras or guide critical steps like suturing, reducing the cognitive load on surgeons and enhancing the accuracy of procedures [82]. Integrating AI-driven assistance in neonatal procedures, such as automating certain suturing steps or providing real-time anatomical guidance, could significantly improve outcomes [83]. Enhanced visualisation technologies, such as augmented reality (AR), can provide real-time overlays of critical anatomical structures, improving the surgeon’s ability to navigate complex anatomical fields. AI-based image enhancement can also help identify and differentiate between tissues in real-time, which is particularly useful in neonates where small size and similar tissue densities pose significant challenges. Future developments should focus on integrating high-resolution, lightweight 3D imaging systems adapted for small operative fields [84]. Integrating AI and AR with advanced robotic systems could enable remote and tele-surgical care in paediatrics. Combined with modular platforms, these technologies would allow expert surgeons to assist remotely, expanding access to specialised care in resource-limited settings.

### Economic and logistical barriers

The high upfront cost of robotic systems, combined with the need for maintenance, specialised equipment and training, poses significant economic barriers to their widespread adoption in paediatric centres [85]. Shanahan (2022) emphasises that consumables alone contribute to over 60% of the cost difference between robotic and non-robotic approaches, highlighting a critical area for cost reduction and an opportunity to develop instruments with longer lifespans to support economic feasibility [86]. Logistical challenges, such as the setup and docking of the robotic system for paediatric use, also present challenges. The average setup time for robotic surgery in neonates, including port placement and docking, is approximately 21.1 min, inviting the development of more user-friendly robotic systems with simplified setup processes and better integration with existing surgical workflows [70]. One potential solution to the economic and logistical challenges is the adoption of modular robotic platforms that can be used across multiple surgical disciplines, which might allow resource sharing and overall implementation cost reduction. Regarding training and adoption, incorporating haptic feedback and AI functionalities into these systems might reduce the learning curve for surgeons, decreasing the amount of specialised training required to adopt robotic technologies. As highlighted by Shanahan (2022), targeted training programs focusing on both technical proficiency and the unique aspects of neonatal surgery are essential to ensure that paediatric surgeons can utilise these advanced systems safely and effectively [86].

### Limitations to this study

The primary limitation of this review is the scarcity of neonatal-specific evidence in robotic cardiothoracic surgery, which means feasibility can only be evaluated conceptually at this stage. Although narrative reviews may introduce selection bias, this was mitigated through a broad multi-database search, predefined inclusion criteria, and the incorporation of all available study types to ensure representative coverage. Published STS-related reports currently offer no clearly identifiable neonatal or infant robotic congenital cases, which limits comparison with established national outcomes; however, this reflects the early developmental stage of the field rather than a methodological constraint. These limitations collectively point toward significant opportunities for future model development, neonatal-specific instrumentation, and translational research to advance robotic neonatal cardiothoracic surgery.

### Conclusion

Robotic-assisted surgery continues to deliver strong advantages in precision, visualisation, and instrument control across multiple specialties, yet its translation to neonatal cardiothoracic surgery remains limited by current constraints in instrument size, articulation, visualisation, and cardiopulmonary tolerability. While present systems, including the Da Vinci 5, do not meet neonatal requirements, ongoing progress in miniaturisation, haptic sensing, AI-assisted navigation, modular architectures, and neonatal-specific simulation offers a realistic path toward future feasibility. Continued innovation, together with targeted model development and engineering–surgical collaboration, will be essential to advance this field. Accordingly, RAS should be viewed not as an immediate clinical option for neonatal CHD, but as a credible and evolving frontier with significant potential for safe and precise application as next-generation platforms mature.

## Supplementary Information

Below is the link to the electronic supplementary material.

Supplementary Material 1

Supplementary Material 2

## Data Availability

No datasets were generated or analysed during the current study.
