# Supplementary material for: Exploring the future of robotic approaches in neonatal cardiac surgery: opportunities, barriers, and innovation pathways
Source: J Robot Surg. 2025 Nov 29;20(1):45. doi: 10.1007/s11701-025-03004-x (PMC12662858; doi:10.1007/s11701-025-03004-x)
Supplement: Supplementary file 2 — Supplementary Material 2 [file 11701_2025_3004_MOESM2_ESM.pdf]

Figure 1

pmc.ncbi.nlm.nih.gov/articles/PMC6469300/figure/dyz009-F3/

board Passmedicine PassMedicine Med school - Goo... P YEAR STUDY SC... Core Conditions Li... CCAS / OSCEs - G... Pharmacology MI...

View full-text article in PMC

Int J Epidemiol. 2019 Feb 19;48(2):455–463. doi: 10.1093/ije/dyz009

Copyright and License information

© The Author(s) 2019. Published by Oxford University Press on behalf of the International Epidemiological Association.

This is an Open Access article distributed under the terms of the Creative Commons Attribution License (http://creativecommons.org/licenses/by/4.0/), which permits unrestricted reuse, distribution, and reproduction in any medium, provided the original work is properly cited.

PMC Copyright notice

Figure 3.

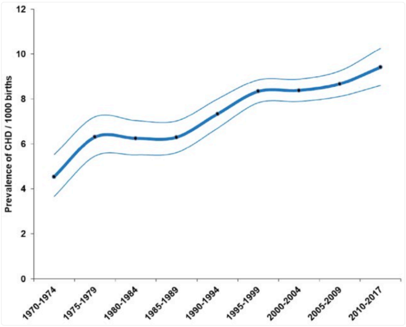

Changes in the birth prevalence of CHD 1970–2017. The thick line is the estimated overall prevalence of CHD, thin lines represent the 95% CI.

Figure 2

pmc.ncbi.nlm.nih.gov/articles/PMC6469300/figure/dyz009-F4/

Passmedicine PassMedicine Med school - Goo... P YEAR STUDY SC... Core Conditions Li... CCAS / OSCEs - G... Pharmacology MI...

An official website of the United States government Here's how you know

in government funding, the information on this website may not be up to date, transactions submitted via the website may not be processed, and the agency may not be able to respond to inquiries until appropriatio
ital of NIH) is open. For more details about its operating status, please visit cc.nih.gov. Updates regarding government operating status and resumption of normal operations can be found at opm.gov.

View full-text article in PMC

Int J Epidemiol. 2019 Feb 19;48(2):455–463. doi: 10.1093/ije/dyz009

Copyright and License information

© The Author(s) 2019. Published by Oxford University Press on behalf of the International Epidemiological Association.

This is an Open Access article distributed under the terms of the Creative Commons Attribution License (http://creativecommons.org/licenses/by/4.0/), which permits unrestricted reuse, distribution, and reproduction in any medium, provided the original work is properly cited.

PMC Copyright notice

Figure 4.

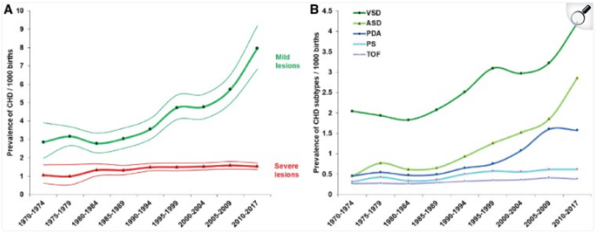

The birth prevalence of CHD subtypes and their changes over time. (A) The prevalence of mild and severe CHD lesions during 1970–2017. Thick lines are the estimated prevalence of CHD lesions, thin lines represent the 95% CI. (B) The birth prevalence of the five most frequent CHD subtypes during 1970–2017. PS, pulmonary stenosis; TOF, tetralogy of fallot.

Figure 4

20/10/2025, 15:58 Re: Permission to reuse content - Siena Martin - Outlook

Share to Teams Zoom

Re: Permission to reuse content

LB

Luca Bertolaccini<luca.bertolaccini@gmail.com>

To: Siena Martin

Mon 10/20/2025 3:21 PM

Cc: pm@amegroups.com; Omar Shafi; Mirkomol Mirzarakhimov <mirkomolmirz@gmail.com>

CAUTION: This email originated from outside of the organisation. Do not click links or open attachments unless you recognise the sender and believe the content to be safe.

Dear Ms. Martin,

Thank you very much for your message. I am pleased to grant permission for the re-use of Figure 5 (“VATS approach in oesophageal atresia. VATS, video-assisted thoracic surgery”) from the article “Approaches to the thoracic cavities” (Bertolaccini L, Pardolesi A, doi: 10.21037/pm.2019.03.06) in your forthcoming narrative review on neonatal cardiothoracic surgery.

Please ensure that the figure is appropriately cited with the following acknowledgment:

“Reproduced from Bertolaccini L, Pardolesi A. Approaches to the thoracic cavities. Pediatric Medicine 2019;3:6. doi: 10.21037/pm.2019.03.06, with permission from the authors.”

I wish you every success with your publication.

With kind regards,

Luca

On 20 Oct 2025, at 16:10, Siena Martin <m2301142@citystgeorges.ac.uk> wrote:

Hello,

Hope you are well. We are writing to request permission to re-use **Figure 5** VATS approach in oesophageal atresia. VATS, video-assisted thoracic surgery., in the following paper: **'Approaches to the thoracic cavities' by Luca Bertolaccini and Alessandro Pardolesi** (doi: 10.21037/pm.2019.03.06). This is for potential use in a narrative review relating to neonatal cardiothoracic surgery.

Grateful for your help and response.

Kind regards,

Siena Martin

Reply Reply all Forward
